# Adaptor CAR Platforms—Next Generation of T Cell-Based Cancer Immunotherapy

**DOI:** 10.3390/cancers12051302

**Published:** 2020-05-21

**Authors:** Claudia Arndt, Frederick Fasslrinner, Liliana R. Loureiro, Stefanie Koristka, Anja Feldmann, Michael Bachmann

**Affiliations:** 1 Institute of Radiopharmaceutical Cancer Research, Helmholtz-Zentrum Dresden-Rossendorf (HZDR), 01328 Dresden, Germany; c.arndt@hzdr.de (C.A.); l.loureiro@hzdr.de (L.R.L.); s.koristka@hzdr.de (S.K.); a.feldmann@hzdr.de (A.F.); 2 Tumor Immunology, University Cancer Center (UCC), University Hospital Carl Gustav Carus, Technical University Dresden, 01307 Dresden, Germany; 3 Medical Clinic and Polyclinic I, Medical Faculty, University Hospital Carl Gustav Carus, Technical University Dresden, 01307 Dresden, Germany; Frederick.Fasslrinner@uniklinikum-dresden.de; 4 National Center for Tumor Diseases (NCT), Carl Gustav Carus, Technical University Dresden, 01307 Dresden, Germany; 5 German Cancer Consortium (DKTK), Partner Site Dresden and German Cancer Research Center (DKFZ), 69120 Heidelberg, Germany

**Keywords:** chimeric antigen receptor (CAR), adaptor molecule, adoptive T cell therapy, cancer immunotherapy

## Abstract

The success of conventional chimeric antigen receptor (CAR) therapy in the treatment of refractory hematologic malignancies has triggered the development of novel exciting experimental CAR technologies. Among them, adaptor CAR platforms have received much attention. They combine the flexibility and controllability of recombinant antibodies with the power of CARs. Due to their modular design, adaptor CAR systems propose answers to the central problems of conventional CAR therapy, such as safety and antigen escape. This review provides an overview on the different adaptor CAR platforms available, discusses the possibilities and challenges of adaptor CAR therapy, and summarizes the first clinical experiences.

## 1. Introduction

Cancer immunotherapy is a rapidly growing field that is becoming more and more important in clinical practice. The development of chimeric antigen receptor (CAR) T cells, formerly known as T-bodies [1], revolutionized adoptive cell transfer. To date, over 520 clinical trials have emerged worldwide, redirecting CAR T cells against 64 different tumor targets [2]. Among them, two CD19-specific CAR T cell products are approved for the treatment of acute lymphoblastic leukemia (ALL) and large B cell lymphoma [3,4,5]. Except for CD19 CARs, the most promising results have currently been achieved for the targeting of CD22 [6] and B cell maturation antigen (BCMA) [7,8] in ALL and multiple myeloma, respectively. However, driven by the selective pressure of mono-specific CAR T cells, antigen-negative escape variants frequently occur and often impede the initial success of these living drugs (e.g., [6,9,10,11,12]). The overall translation of CAR technology to non-hematologic malignancies remains challenging. Physical barriers and the immunosuppressive microenvironment of solid tumors represent major obstacles, as they impair CAR T cell migration and function [13]. Low levels of target antigen expression in healthy tissues can result in severe “on-target, off-tumor” toxicities, as exemplified by the occurrence of lethal pulmonary toxicity in a colon cancer patient after the infusion of autologous *α*-HER2/neu CAR T cells [14]. Besides the damage of healthy tissues, cytokine release syndrome (CRS) and CAR T cell-related encephalopathy syndrome (CRES) are frequently observed side effects of CAR T cell therapy [15].

In order to overcome the above-mentioned limitations, the scientific community is pursuing various new CAR concepts and models [13]. This review will focus on recent advances in the field of switchable adaptor CAR platforms, with emphasis on their safety profile, controllability, target flexibility, specificity, and efficiency.

## 2. Adaptor CAR Design

**Figure 1:**
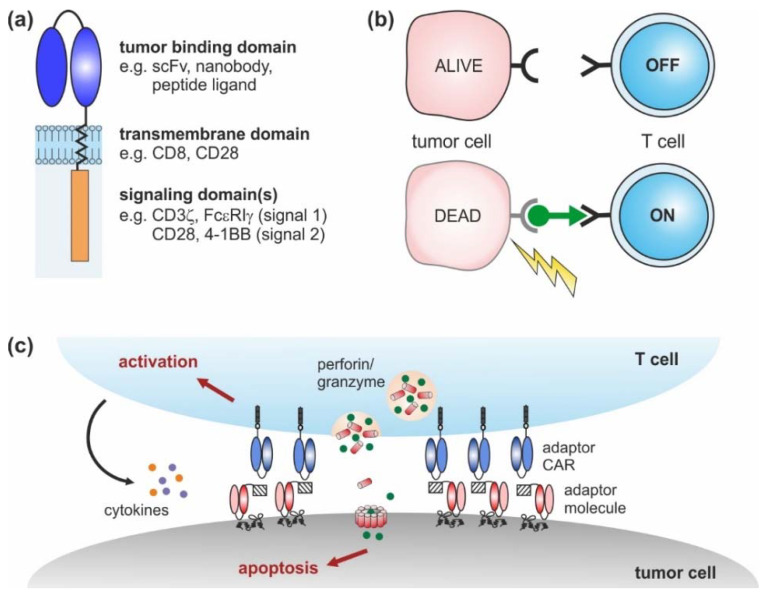
Chimeric antigen receptor (CAR) design: (**a**) conventional CARs are composed of an extracellular tumor binding domain followed by a transmembrane and intracellular signaling domains. (**b**) Adaptor CAR systems are composed of T cells engineered with an adaptor CAR and soluble adaptor molecules (green). Adaptor CAR T cells are per se inactive (OFF). In the presence of tumor-specific adaptor molecules, they are turned ON. (**c**) Upon cross-linkage via adaptor molecules, adaptor CAR T cells elicit potent anti-tumor responses that finally result in tumor lysis.

CARs are artificial receptors composed of three key elements: an extracellular (tumor binding) domain followed by a transmembrane and intracellular signaling domain(s) (Figure 1a) [16,17]. Similar to antibodies, CAR-modified T cells are able to bind naturally occurring surface molecules independent of their own T cell receptor (TCR). Upon tumor recognition, downstream signaling pathways are activated, triggering the lysis of malignant cells [16,17]. Over the last decades, CAR design has undergone continuous changes from 1st to 4th generation that have improved CAR T cell expansion, cytotoxicity, cytokine secretion, and in vivo persistence [18].

Adaptor CAR T cells were developed with the aim to improve the flexibility, tumor specificity, and controllability of conventional CAR T cells. To achieve these objectives, the tumor-targeting and signaling moieties of conventional CARs were uncoupled, resulting in a dichotomous system consisting of an adaptor CAR and soluble, tumor-specific adaptor molecules (Figure 1b). The basic structure of adaptor CARs corresponds to the conventional CAR design (Figure 1a), although the extracellular domain does not interact with a tumor-associated antigen but with a binding partner incorporated into the adaptor molecule. The bifunctional adaptor molecule in turn provides tumor specificity and acts as a linker at the interface between the tumor and the adaptor CAR T cell. This complex can then mediate anti-tumor responses, similar to conventional CAR T cells (Figure 1c).

Notably, the dual principle of adaptor CAR systems provides an important molecular safety switch to precisely control the adaptor CAR T cell activity. Anti-tumor responses will decrease and vanish with the elimination of the adaptor molecule from the body. Vice versa, repeated adaptor molecule administration will permit the re-initiation of therapy against the same or an alternative target in case of tumor relapse. Overall, the adaptor CAR T cell activity as well as the associated side effects (e.g., CRS, “on-target, off-tumor” toxicity) might be controlled in a time- and dose-dependent manner, which is an important step towards precision medicine. The switch control mechanism of adaptor CARs clearly deviates from current clinical practice to manage CAR T cell-related side effects—e.g., the use of corticosteroids that systemically suppress the entire immune system. Alternative strategies to improve safety—e.g., the integration of suicide genes (inducible Fas or caspase 9, herpes simplex virus thymidine kinase), or elimination genes (CD20, epidermal growth factor receptor (EGFR))—result in CAR T cell depletions that entail the irreversible loss of the costly cell products [19,20,21,22,23,24,25,26]. The controllability of the adaptor CAR therapy will be addressed in Section 3 in more detail.

Due to their dichotomous nature, adaptor CAR platforms overcome the rigid mono-specificity of conventional CARs. This flexibility offers novel possibilities to encounter one of the central problems of conventional CAR T cell therapy—the antigen-negative relapse. One adaptor CAR can redirect T cells against a theoretically unlimited number of target antigens. Thus, the technology allows the manufacturing of one bioengineered T cell product universally applicable for all types of cancer. This obviates the need for the laborious and cost-intensive development of new CAR constructs and genetically modified immune cells. Provided that a comprehensive library of appropriate adaptor molecules is available, adaptor CAR T cells can be easily used for simultaneous or consecutive multiple tumor targeting. This aspect and target specificity of adaptor CAR therapy will be discussed in detail in Section 4.

**Figure 2:**
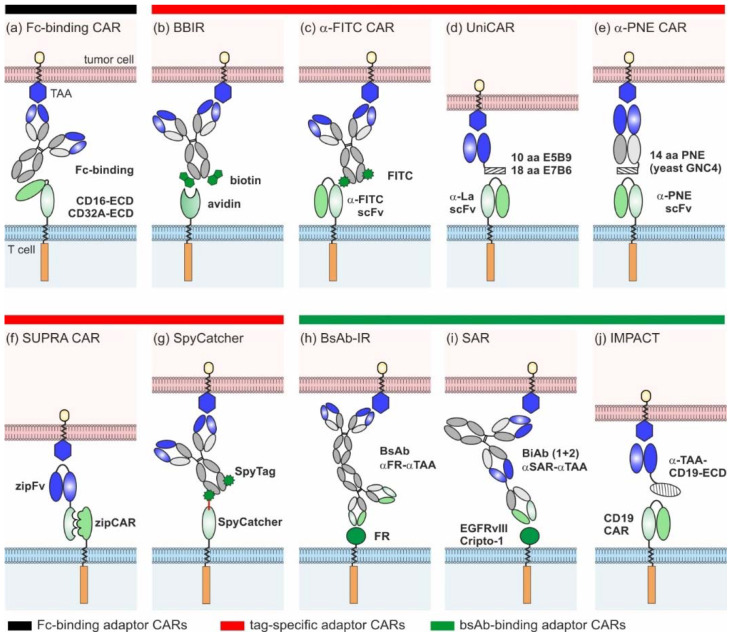
Overview of different adaptor CAR designs: (**a**) Fc-binding CAR, (**b**) biotin-binding immune receptors (BBIRs), (**c**) *α*-FITC CARs, (**d**) UniCARs, (**e**) *α*-peptide-neoepitope (PNE) CARs, (**f**) split, universal, and programmable (SUPRA) CARs, (**g**) SpyCatcher, (**h**) bispecific antibody-binding immune receptors (BsAb-IRs), (**i**) synthetic agonistic receptors (SARs), and (**j**) Integrated Modules oPtimize Adoptive Cell Therapy (IMPACT).

Since 2006, several groups have devised ten different adaptor CAR platforms that can be subdivided into three major classes (overview Figure 2):

1. Fc-binding adaptor CARs;
2. Tag-specific adaptor CARs;
3. Bispecific antibody (bsAb)-binding adaptor CARs.

### 2.1. Fc-Binding Adaptor CARs

Clémenceau and colleagues published the basic idea of adaptor CARs in 2006 [27]. They took advantage of the well-investigated interaction between CD16 and the Fc-part of IgG molecules and constructed the first adaptor CAR composed of the CD16 extracellular domain (ECD) and FcεRIγ intracellular-signaling domains [27]. Since then, the CD16 adaptor CAR platform was further refined by the construction of different alternative CD16 CARs of the 1st [28,29] and 2nd generation [30,31]. Only in combination with tumor-specific monoclonal antibodies (mAb) (e.g., rituximab, trastuzumab, cetuximab), CD16 CAR T cells triggered efficient tumor lysis both in vitro and in vivo (Figure 2a) [27,28,29,30,31]. Due to the low binding affinity of CD16 to human IgG2, Caratelli et al. later utilized the CD32A ECD for the construction of adaptor CARs that are able to bind IgG1 and IgG2 with similar affinities [32]. The therapeutic activity of antibodies is clearly determined by their glycosylation pattern [33]. Accordingly, glyco-engineered antibodies were shown to amplify CD16 CAR T cell activity [31]. As this might entail an increased risk of severe side effects (e.g., CRS), suitable adaptor mAbs need to be assessed carefully [31]. A major advantage of Fc-binding adaptor CAR systems is the availability of a wide repertoire of antibody-dependent cellular cytotoxicity (ADCC)-mediating mAbs in clinical grade quality [34], circumventing extensive antibody engineering and easily enabling target switch during therapy. Although therapeutic effects might be hampered by interference with naturally occurring IgG molecules, it was shown that an excess of irrelevant immunoglobulins did not inhibit but rather increased the cytotoxic capacity of the CD16 adaptor CAR systems [30,31]. The authors assume that the unspecific deposition of IgG on cancer cells was responsible for enhanced killing and thus recommend excluding patients with IgG-associated pathologies from CD16 CAR therapy [31].

### 2.2. Tag-Binding Adaptor CARs

In general, tag-specific adaptor CARs harbor an ECD that recognizes a chemically, enzymatically, or genetically attached tag of tumor-specific adaptor molecules.

Biotin-binding immune receptor (BBIR) T cells were the first tag-specific adaptor CARs described in the literature (Figure 2b) [35]. They harness the highly specific, non-covalent interaction of avidin or streptavidin with biotin. The 67 kDa chicken avidin and the 53 kDa bacterial streptavidin (*Streptomyces avidinii*) can bind up to four biotin molecules simultaneously. Based on these proteins, different BBIRs were designed, carrying either monomeric or dimeric biotin-binding molecules as ECDs [35,36]. In combination with biotinylated mAbs or single-chain fragment variables (scFvs), only dimeric chicken avidin (dcAv) CAR T cells and monomeric streptavidin (mSA2) CAR T cells were proven to be useful tools for the in vitro and in vivo targeting of various cancer cells overexpressing, e.g., epithelial cell adhesion molecule (EpCAM), EGFR, and CD20 [35,36]. As soluble biotin did not inhibit the performance of BBIR CAR T cells, the risk of interference with biotin naturally present in patients seems to be low [35,37]. However, the occurrence of natural anti-biotin antibodies in human serum [38] and the antigenicity of avidin and streptavidin (e.g., [39,40]) might interfere with therapeutic effects. Whether this can induce undesirable adverse reactions or hamper the successful clinical translation of the BBIR adaptor system has yet to be investigated. 

Another semi-synthetic adaptor CAR system relies on scFv-based *α*-FITC CARs targeting the synthetic dye fluorescein isothiocyanate (FITC) that is chemically coupled to various tumor-specific adaptor molecules (Figure 2c) [41]. The first proof of concept studies by Tamada and colleagues demonstrated that T cells carrying 3rd generation *α*-FITC CARs are able to elicit potent anti-tumor responses in the presence of FITC-labeled cetuximab, rituximab, and trastuzumab [41,42,43,44,45,46,47,48,49]. Over time, different classes of FITC-conjugated adaptor molecules were studied, including targeting compounds based on fragments of antigen binding (Fabs) [43,44] and small molecules [42,45,46,47,48,49]. As shown for HER2- and CD19- and CD20-Fabs, the position and stoichiometry of the FITC label influenced the *α*-FITC CAR T cell activity [43,44]. The consequential need for the individual optimization of each target contradicts the fast adaptability of adaptor CAR platforms. The most widely studied adaptor molecule is EC17, a folate-FITC conjugate [42,46,47,48,49] initially designed and clinically tested (ClinicalTrials.gov. Identifier: NCT01996072, NCT01994369, NCT02000778, NCT01778933, NCT01778920, NCT01511055, NCT00485563) for the image-guided surgery of inoperative solid tumors (e.g., [50]). The small molecular weight adaptor efficiently redirected the *α*-FITC CAR T cells against different tumor entities—e.g., non-small lung cancer, breast cancer, and osteosarcoma—allowing for controllability in a time- and dose-dependent manner [46,47,48,49]. Despite the full humanization of the *α*-FITC CAR T cell product [44], the immunogenic potential of FITC is one concern for clinical translation, as underlined by the emergence of *α*-FITC antibodies in therapeutic mouse models [41].

Alternatively, adaptor molecules can be endowed with small peptide tags to redirect standard scFv-based adaptor CARs. The UniCAR platform introduced in 2014 [51,52] utilizes the 10 amino acid (aa) peptide epitope E5B9 derived from the human nuclear La/SS-B protein [53,54]. Meanwhile, a broad library of E5B9-tagged adaptor molecules, so-called target modules (TMs), were developed to specifically cross-link UniCAR T cells with tumor cells (Figure 2d). They were built on different binding moieties (small peptide molecules, nanobodies, and scFvs), targeting various antigens overexpressed in hematologic and solid tumors (e.g., CD19, CD33, CD123, CD98, EGFR, disialoganglioside (GD2), prostate stem cell antigen (PSCA), prostate-specific membrane antigen (PSMA), and sialyl-Tn (STn)) [52,55,56,57,58,59,60,61,62,63]. Recently, larger IgG-based TMs were effectively used for the redirection of UniCAR T cells against GD2- and STn-expressing cancer cells in vitro and in vivo [64,65]. The anti-tumor responses were comparable to small scFv-based TMs, but the serum half-lives considerably increased, which will impact future dosing regimens (see Section 3). Apart from E5B9, the 18 aa *α*-helical La epitope E7B6 was also successfully employed in the UniCAR system [66]. Although La/SS-B is a well-known autoantigen, the immunogenic potential of E5B9 and E7B6 is expected to be very low, as both epitopes are cryptic in the native La protein and none of the La-specific antibodies found in the sera of >100 autoimmune patients showed reactivity against these epitopes [67,68,69,70,71]. To reduce the overall immunogenicity of UniCAR components, the *α*-La and tumor-specific scFvs of UniCARs and TMs were humanized, respectively. Similarly to the UniCAR approach, scFv-based *α*-peptide neo-epitope (PNE) CARs recognize adaptor molecules endowed with a 14 aa peptide epitope derived from the yeast transcription factor GNC4 (Figure 2e) [43,72,73,74]. As this PNE is not naturally occurring in humans, it is at greater risk of inducing immune responses in patients. All the PNE-tagged adaptor molecules described so far were constructed based on tumor-specific IgG molecules or Fabs, while the latter were preferred due to their favorable pharmacokinetic properties [72]. PNE-tagged Fabs against CD19, CD20, and HER2 effectively redirected adaptor CAR T cells for the lysis of B cell lines [72] and breast cancer cell lines [43], as well as patient-derived pancreatic cancer cells [73]. The *α*-PNE CAR T cell activity and phenotype was temporarily controlled via adaptor molecule dosing [72,74]. Interestingly, the modifications of *α*-PNE CAR hinge region, PNE conjugation site, and number considerably altered the geometry of the immunological synapses and thereby influenced the overall performance of the *α*-PNE adaptor CAR T cells [72]. The best adaptor molecule designs were dependent on the selected tumor antigen [43,72], as was also observed for FITC-labeled Fab-switches [43,44].

In 2018, Cho et al. introduced the split, universal, and programmable (SUPRA) CAR technology (Figure 2f), which utilizes leucine zippers as interaction partners between adaptor CAR T cells and adaptor molecules [75]. The so-called zipCAR was designed by the fusion of a leucine zipper to the intracellular signaling domains of 4-1BB and CD3ζ. ZipFvs function as adaptor molecules and are composed of tumor-specific scFvs (*α*-HER2, *α*-AXL, *α*−mesothelin) and a cognate leucine zipper. The amphipathic interactions between two ZIP domains determine the zipCAR/zipFv affinities that are exploited to fine-tune the specificity and activity of zipCAR T cells (see Section 3 and Section 4). In addition to synthetic leucine zippers, leucine zippers derived from human FOS and JUN were used to create a humanized SUPRA CAR system.

SpyCatcher immune receptors are a novel class of adaptor CARs. Unlike others, they are able to covalently bind SpyTag-containing adaptor molecules (Figure 2g) [76]. The SpyTag/SpyCatcher system originated from the immunoglobulin-like collagen adhesion domain of *Streptococcus pyogenes* (CnaB2), which contains an internal isopeptide bond between aa position 31 (Lys) and 117 (Asp) [77]. The separation of CnaB2 and subsequent modifications resulted in the Lys31-containing SpyTag peptide (13 aa) and Asp117-containing SpyCatcher protein (116 aa) [77]. Both binding partners first associate non-covalently with a high affinity, rapidly followed by a spontaneous, autocatalytic isopeptide bond formation between Lys31 and Asp117 [77]. To create 2nd generation SpyCatcher CARs, the SpyCatcher protein was connected with the intracellular CD3ζ and CD28 or 4-1BB signaling domains [76]. The SpyTag in turn was genetically fused or site-specifically attached to HER2-, EGFR-, EpCAM-specific Designed Ankyrin Repeat Proteins (DARPins), and clinical-grade IgG molecules (rituximab, trastuzumab, cetuximab) [76]. In the first proof of concept studies, Minotulu et al. [76] demonstrated that SpyTag-containing adaptor molecules were efficiently attached to SpyCatcher-immune receptor-equipped T cells and subsequently mediated efficient tumor cell lysis in vitro and in vivo. Upon antigen-specific stimulation, preloaded SpyCatcher immune receptors are internalized, ensuring an off-switch. Thus, adaptor CAR T cells lose their target specificity over time and require continued rearming with SpyTag-containing adaptor molecules. The possibility of covalently arming SpyCatcher adaptor CAR T cells with one or multiple target specificities prior to infusion is a unique feature of this system.

### 2.3. BsAb-Binding Adaptor CARs

Due to their dual specificity for a tumor-specific antigen and an activating immune receptor (e.g., CD3), bsAbs are able to redirect T cells for highly efficient tumor cell killing [78]. In 2014, Urbanska and colleagues conceived the idea to combine the power of bsAbs with CARs; they developed the first bsAb-binding immune receptor (bsAb-IR), comprising the extracellular part of human folate receptor *α* (FR*α*) (231 aa) and 1st or 2nd generation CAR signaling domains (Figure 2h) [79]. Bispecific adaptor molecules were created by the chemical heteroconjugation of *α*-FR*α* and *α*-CD20 mAbs. Although this was the first in vitro data to verify the general functionality of the system, the lytic activity of the redirected FR*α* CAR T cells against B cell lines was low due to the poor quality of the bsAb adaptors [79]. Later, Karches et al. presented alternative bsAb-binding adaptor CARs containing the ECD of human epidermal growth factor receptor variant III (EGFRvIII) or human Cripto-1 and termed them synthetic agonistic receptors (SARs) (Figure 2i) [80]. In their studies, they explored both tetravalent (2 + 2) and trivalent (2 + 1) bispecific adaptors targeting EpCAM or mesothelin in murine and human mouse models. Data have proven that only bsAbs with one binding arm for the SAR-ECD are able to trigger T cell activation, proliferation, and tumor lysis in a strictly target-dependent manner. To avoid cross-reactivity with healthy tissues, the ECD of bsAb-binding adaptor CARs should be carefully selected. As EGFRvIII is exclusively expressed in malignant cells and Cripto-1 is an embryonic antigen, they possess a relatively low risk of unwanted side effects.

In 2017, Ambrose and colleagues introduced the IMPACT (Integrated Modules oPtimize Adoptive Cell Therapy) strategy (Figure 2j) [81,82]. They refashioned conventional CD19 CAR T cells into adaptor CARs. Bifunctional fusion proteins, which are composed of an optimized variant of the CD19-ECD and a tumor-specific binding moiety (e.g., scFv), served as bridging molecules between the tumor and CD19 CAR T cells [81,82,83]. By using this approach, the CD19 CAR T cells elicited potent anti-tumor responses in experimental mice models—e.g., against CD19^neg^HER2^pos^ and CD19^neg^CD20^pos^ tumors. The adaptor molecules were delivered either via infusion or directly via CD19 CAR T cells [81,82,83]. To achieve the latter, lentiviral constructs encoding the CD19 CAR and the adaptor molecule were designed. Overall, the IMPACT strategy is a promising method to repurpose CD19 CAR T cells for targeting alternative tumor-associated antigens after CD19^neg^ disease relapse, such as CD20 [83]. Although the versatility of the systems was proven for solid tumors [82], the risk of CD19 CAR T cell therapy-related side effects such as CRS, CRES, and B cell aplasia remains and impairs the safety profile of this approach.

## 3. Controlling Therapy-Related Side Effects with Adaptor CARs

Adaptor molecules are the key element for controlling adaptor CAR T cell activity. The on/off-switch rate is mainly determined by their pharmacokinetic properties and biodistribution, as well as their binding affinities towards the target antigen and the ECD of adaptor CARs.

**Figure 3:**
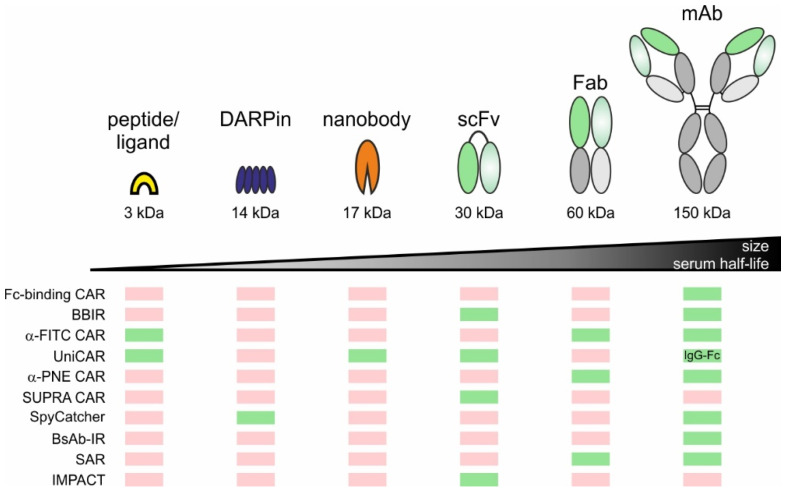
Binding moieties used for adaptor molecule design. Overall, small peptides/ligands, Designed Ankyrin Repeat Proteins (DARPins), nanobodies derived from camelid antibodies, single-chain fragment variables (scFvs), fragments of antigen binding (Fabs), and full size monoclonal antibodies (mAbs) were used to engineer adaptor molecules. Boxes below indicate whether or not the binding moiety was already applied to a certain adaptor CAR platform (green: yes; red: no).

The targeting moieties used for adaptor molecule design range from small peptide/receptor ligands (3 kDa), DARPins (14 kDa), nanobodies (17 kDa), and scFvs (30 kDa) to larger Fabs (60 kDa) and mAbs (150 kDa) (Figure 3). The influence of the adaptor molecule size on in vivo pharmacokinetics and -dynamics were studied using positron emission tomography (PET) imaging [57,58,60,62,64,65,84]. Peptide ligand-, nanobody-, and scFv-based adaptor molecules smaller than 60 kDa were rapidly cleared via the kidneys, with serum half-lives between 20 to 90 min [48,57,58,60,62,84]. In contrast, larger IgG-based TMs (115 kDa) showed extended serum half-lives of 12–39 h [64,65], similar to Fabs (12–24 h) [85]. Monoclonal Abs possess by far the highest serum half-life of 10 d [86]. In general, smaller adaptor molecules are expected to enable better temporal control due to their fast elimination but, on the other hand, require permanent infusions to maintain anti-tumor activities. Consequently, using small adaptor molecules in the early treatment phase has the advantage that potential side effects could be stopped immediately simply by discontinuing their infusion. The possibility for such a rapid on/off-switch is mainly important to control acute side effects such as CRS. In contrast, the application of large molecules will delay safety management as they may circulate for weeks in the body [86]. To preclude any complications, large adaptor molecules should be favored when the risk for acute side effects is low. In case of acute toxicities, a rapid off-switch also requires the fast disassembling of adaptor molecules from tumor and adaptor CAR T cells. Based on dynamic PET data, Albert et al. showed that the serum half-life of nanobody-based *α*-EGFR TMs slightly increased from 1.7 h to 7 h and 19.4 h for UniCAR- and tumor-bound TMs, respectively [57]. Although the elimination was delayed, the cell-bound adaptors still rapidly cleared from the blood within only a few hours in contrast to large mAbs, which are detectable for several days or even weeks in serum.

As CAR T cell therapy-induced cytokine release is not only a life-threatening risk for patients, but at lower levels also indicative of a successful engraftment and therapy response [47], a good compromise between adaptor CAR T cell activation and deactivation is pivotal. To address this question, tumor growth and cytokine release were monitored dependent on the applied adaptor molecule dose in experimental mice [44,47,72,75]. Thereby, different dosing regimens that mitigated treatment-related adverse reactions without the loss of adaptor CAR T cell cytotoxicity were identified. In an immunodeficient Nalm-6 model, the low dosing of PNE-tagged CD19-Fab adaptors (0.05 mg/kg) was sufficient to achieve complete tumor remission [72]. Although the tumor clearance was delayed compared to conventional CD19 CAR T cells, the cytokine release was kept at low levels [72]. Other studies demonstrated that treatment-related toxicities of *α*-FITC and *α*-PNE CAR T cells were minimized by escalating the adaptor molecule dosing, while anti-tumor responses remained high [44,47,72]. In addition, the CRS-like symptoms observed in mice were successfully controlled by interrupting continuous adaptor molecule infusions [44]. Interestingly, the highest efficiency with the best safety profile was achieved with either a slow dose escalation or a reduction in adaptor infusion frequency to one dose per week [44]. Considering the short serum half-life of the applied low molecular weight adaptor folate-FITC (EC17) [87], one single dose per week was not expected to be superior to continuous infusions. Thus, the results indicate that overstimulation and associated T cell exhaustion together with the high level of cytokine-induced toxicities might counteract successful treatment. This is in line with previous findings of *α*-PNE adaptor CARs showing that low adaptor doses and interrupted dosing regimens (1–2 weeks rest phase) correlated with increased proportions of central memory CAR T cells [72,74].

The termination of adaptor molecule dosing is the most effective approach to permanently turn off adaptor CAR T cell activity and face the long-term destruction of target antigen-expressing healthy tissues. In a fully murine B cell depletion model, Ma et al. demonstrated that B cells repopulated within a short time frame after the termination of CD19-directed FITC adaptor infusion [44]. Several groups further confirmed the functional reversibility of adaptor CAR systems in immunodeficient or competent mouse models, underlining that therapeutic effects can be rapidly re-initiated to control tumor growth in case of tumor relapse (e.g., [44,72,74,80]). In a clinical setting, rapidly eliminated adaptors will facilitate a prompt intervention and fast manipulation of adaptor molecule doses. This is especially important at the beginning of therapy, when the tumor burden and risk of acute toxicities is high. Large adaptor molecules with prolonged serum half-lives can only be regulated with considerable delays, and thus should be favored to maintain tumor remission later in the course of therapy, when potential side effects are known to be tolerated. In order to further accelerate the off-switch of engaged adaptor CAR T cells, the deprivation of adaptor molecules might be combined with the application of competing compounds. Studies with *α*-FITC-, SUPRA-, and UniCAR T cells have shown that competitor molecules diminish cytokine secretion and tumor elimination and can release/block adaptor molecules from tumor binding [41,42,47,49,62,75]. Several different agents were employed, including non-specific adaptor molecules [41]; non-tagged targeting ligands, such as folate [47,49] and 2-(phosphonomethyl)-pentandioic acid (2-PMPA) [62]; free adaptor tags, e.g., fluorescein [42,47]; and adaptors with cognate binding partners [75]. Alternatively, tyrosine-kinase inhibitors might be applied to control side effects temporarily. The co-administration of clinically relevant Midostaurin doses and CD33-directed UniCAR T cell therapy resulted in the considerable inhibition of UniCAR T cell function with regard to T cell proliferation, cytokine secretion, and tumor cell killing in vitro [88]. These observations were not only limited to hematologic tumor models but also confirmed for solid tumors [88].

Besides the application of distinct dosing regimens or blocking agents, adaptor CAR T cell function can be finely regulated via the binding affinities of adaptor molecules towards their cognate receptors [75]. As shown in the SUPRA CAR system, the application of zipFvs with different zipper affinities as well as zipFvs with competitive cognate leucine zippers permitted researchers to gradually control cytokine levels in vivo. By designing orthogonal SUPRA CARs that activate distinct signaling pathways (TCR signaling via CD3ζ or costimulatory signaling via CD28/4-1BB), “AND” gate logical gating could be further used to fine-tune adaptor CAR T cell activation (CD69 expression) and cytokine secretion (IFN-γ) [75].

Although not anticipated with adaptor CAR systems, as a last resort, adaptor CAR T cells can be irreversibly depleted via integrated elimination tags or suicide genes, as pursued for conventional CAR T cells [19,20,21,22,23,24,25,26]. For example, EGFRvIII SAR T cells are in principle prone to Cetuximab-mediated killing [80]. Conversely, adaptor CAR T cells might be used to rescue patients from CAR treatment-related side effects. A prerequisite is the integration of an adaptor-tag, e.g., E7B6, as suggested by the anti-CAR-CAR approach [66]. This strategy would allow conventional CAR T cell therapy to be ceased after remission, thus avoiding long-term “on-target, off-tumor” toxicities, while preserving inert adaptor CAR T cells that could be repurposed for tumor cell killing in case of tumor relapse.

## 4. Improving Treatment Efficiency and Target Specificity

**Figure 4:**
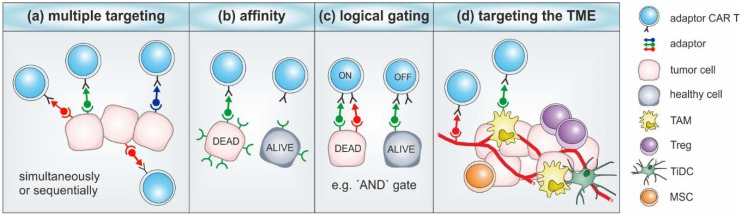
Flexibility and specificity of adaptor CAR systems. (**a**) Adaptor CAR T cells can be redirected either simultaneously or sequentially to multiple targets via different adaptor molecules. (**b**) By modulating the binding affinities, they are able to distinguish target cells with different antigen densities. (**c**) Logical gating strategies can be applied to increase the specificity of adaptor CAR T cells. (**d**) Combined targeting of tumor cells and cellular components of the tumor microenvironment (TME) might boost the efficiency of adaptor CAR therapy. TAM: tumor-associated macrophage; Treg: regulatory T cell; TiDC: tumor-infiltrating dendritic cell; MSC: myeloid suppressor cell.

Multiple tumor targeting is a feasible method to address the heterogeneity of tumors and associated antigen escape. In this regard, adaptor CAR T cells were successfully used for the simultaneous or consecutive targeting of multiple tumor antigens by applying mono- or even multispecific adaptor molecules (Figure 4a) [35,48,55,56,75,76]. Preclinical in vitro studies with the UniCAR system demonstrated that dual-targeting approaches can be even superior to mono-specific treatments with regard to killing efficiency and cytokine production [55,56]. Notably, cocktails of FITC-labeled low molecular weight adaptors enabled *α*-FITC CAR T cells to completely eradicate antigenically heterogeneous tumors in mice, while infusions of only one adaptor molecule were not able to control tumor growth [48].

Although adaptor CAR T cells are switchable systems, it is highly recommended to minimize possible “on-target, off-tumor” toxicities by increasing their target specificity. In order to accomplish discrimination between malignant cells and healthy tissues, adaptor molecules of different affinities can be applied (Figure 4b) [57,60,75]. For instance, EGFR-directed UniCAR T cells were able to discriminate between EGFR^low^ and EGFR^high^ tumor cells in the presence of a monovalent EGFR TM [57,60]. In contrast, the bivalent EGFR-EGFR TM version was not able to distinguish between different antigen densities [57,60]. As exemplified by studies from Cho and colleagues, adaptor CAR T cell specificity can further be logically gated by applying the rules of Boolean algebra (Figure 4c) [75]. In order to achieve “NOT” logical gating, they developed *α*-Axl and *α*-HER2 zipFvs with complementary zippers. In the presence of cells that express both antigens, *α*-Axl and *α*-HER2 zipFvs are able to interact with each other and thus protect healthy cells from SUPRA CAR T cell-mediated killing. An alternative gated targeting strategy was described for *α*-FITC adaptor CARs [45]. In this “AND” gate model, T cells were engineered to express both *α*-FITC-CD3ζ and *α*-mesothelin-4-1BB CAR constructs. As shown by in vitro and in vivo studies, dual receptor CAR T cells only exerted potent anti-tumor responses in the presence of both signals. Thus, dual CAR activity and specificity was controllable via a FITC-labeled bifunctional adaptor molecule (HM3-FITC) [45]. As logically gated adaptor CAR systems have no fixed antigen specificity, they can be easily and safely translated to other tumor entities and target combinations.

The tumor microenvironment (TME) represents one of the major hurdles that needs to be overcome for the successful translation of CAR technologies to solid tumors. Thus, the combined targeting of tumor cells and cellular components of the TME might significantly boost anti-tumor responses (Figure 4d). Attractive candidates are, for example, PSMA, which is known to be overexpressed on the tumor neovasculature [89,90]; hypoxia-induced carbonic anhydrase IX [91]; and folate receptor *β* (FR*β*) for the targeting of tumor-associated macrophages [92]. All of these targets were already successfully addressed by different adaptor CAR systems in the context of tumor cell killing [46,48,56,62]. The feasibility of TME targeting is underlined by the studies of Chu and colleagues [46]. They demonstrated that the small molecular weight adaptor folate-FITC redirected *α*-FITC CAR T cells for the efficient killing of FR*β*^pos^ macrophage cell lines [46]. As exemplified by comparative studies with different UniCAR constructs, intracellular signaling domains might further influence the ability of adaptor CAR T cells to overcome immunosuppression by tumor-infiltrating regulatory T cells (Tregs) [93]. In contrast to UniCAR-4-1BB/ζ T cells, UniCAR T cells with intracellular CD28- and CD3ζ-signaling domains possessed an increased resistance to Treg-mediated immunosuppression that was accompanied by higher cytokine levels. Apart from the hostile TME, (adaptor) CAR T cell therapy might be challenged by treatment responses to previous therapeutic interventions, e.g., chemo- or radioresistance. Just recently, we reported that CD98- and EGFR-redirected UniCAR T cells efficiently eliminated radioresistant head and neck cancer cells in vitro and in vivo [63]. Although high CD98 and EGFR expression is associated with radioresistant phenotypes, both antigens are widely expressed at low levels in non-cancerous tissues. To avoid possible “on-target, off-tumor” effects, including the fratricide of adaptor CAR T cells, “AND” gate-targeting strategies might be desired for clinical translation [63].

## 5. Co-Delivery of Payloads via Adaptor Molecules

The unique design of adaptor CAR systems further allows the easy co-delivery of payloads locally via soluble adaptors. On the one hand, the integration of co-stimulatory molecules, such as 4-1BBL or Ox40L, is a feasible method to ameliorate adaptor CAR T cell-mediated tumor cell killing, as exemplified by a CD123-4-1BBL TM in the UniCAR system [94]. On the other hand, adaptor molecules might be repurposed to co-deliver radionuclides for imaging or internal radiation. In order to create an adaptor molecule with diagnostic and therapeutic potential, we converted the clinically used radiotracer PSMA-11 into a UniCAR TM [62]. This theranostic molecule efficiently redirected UniCAR T cells for tumor cell killing and allowed the visualization of tumor lesions in a prostate cancer patient with high resolution [62]. Likewise, other UniCAR TMs targeting, e.g., GD2 [64], EGFR [57,60], STn [65], and PSCA [84], were successfully radiolabeled and applied for both cancer immunotherapy and tumor visualization in mice. As malignant cells evolve and evade under the selection pressure of (adaptor) CAR T cell immunotherapy, such theranostic adaptor molecules might be promising tools to spatially and temporarily follow this dynamic process. They can be periodically used for target verification and the assessment of molecular therapy responses and thereby help to guide an appropriate patient-specific treatment. Alternatively, adaptor molecules might be used to deliver high therapeutic radiation doses for internal radioimmunotherapy, toxins, or fluorescent dyes for the image-guided surgery of inoperative solid tumors, as exemplified by clinical studies with the adaptor molecule folate-FITC (EC17) (e.g., [50]).

## 6. Clinical Translation of Adaptor CAR Platforms

The first adaptor CAR T cells tested in humans were the antibody-coupled T cell receptor ACTR087 and ACTR707 T cell products (Unum Therapeutics Inc., Cambridge, MA, USA). These CD16 CARs were combined with the mAbs rituximab, SEA-BCMA, and trastuzumab for the treatment of CD20^pos^ B cell lymphoma, BCMA^pos^ multiple myeloma, and HER2^pos^ solid tumors, respectively (Table 1). Results are available for the treatment of CD20^pos^ B cell lymphoma patients and HER2^pos^ solid tumors. In the presence of rituximab, low doses of ACTR087 T cells (0.5 × 10^6^ cells/kg, CD16-BB/ζ) triggered 3/6 complete or partial responses, with no adverse reactions in CD20^pos^ B cell lymphoma patients [95]. However, serious side effects were observed at higher ACTR087 T cell doses (1.5 × 10^6^ cells/kg). Two out of nine CD20^pos^ B cell lymphoma patients experienced fatal treatment-related toxicities including CRS and CRES, which lead to the first temporary hold of the clinical study [95]. After continuation in February 2018, the U.S. Food and Drug Administration (FDA) placed a second hold on the study in July 2019 when Unum therapeutics reported again on a CD20^pos^ B cell lymphoma patient with grade 3 neurotoxicity and grade 4 respiratory distress in the dose-escalation cohort [96]. In January 2020, the enrollment of cohort 1 in a dose-escalating clinical trial evaluating ACTR707 T cells (25 × 10^6^) in combination with trastuzumab (1.0 mg/kg weekly) for the treatment of HER2^pos^ solid tumors (ClinicalTrials.gov identifier: NCT03680560) was successfully completed. Interestingly, here no dose-limiting toxicities occurred [97]. The observed differences compared to the ACTR707-based clinical studies might be explained by the usage of different intracellular signaling domains in the CD16 CAR constructs. While ACTR087 possesses both a CD3ζ and 4-1BB intracellular-signaling domain, ACRT707 is only equipped with the co-stimulatory domain of CD28. It would be also plausible that the divergent safety profiles are related to different glycosylation patterns or isotypes of the selected mAb rituximab and trastuzumab [31,33]. However, the underlying reasons are still not known and need to be investigated in more detail. Besides CD16 CARs, just recently the adaptor CAR platform UniCAR (Cellex Patient Treatment GmbH, Dresden, Germany) entered a phase 1 clinical trial (ClinicalTrials.gov identifier: NCT04230265). In a dose-escalation study, the efficiency of UniCAR02-T cells combined with CD123 TMs will be investigated in patients with CD123^pos^ hematologic and lymphatic malignancies. In February 2020, Calibr and AbbVie received permission from the FDA for clinical trials with *α*-PNE CAR T cells and an *α*-CD19 Fab-based switch (CLBR001 + SWI019) in relapsed/refractory B cell malignancies [98]. Although *α*-FITC CARs (Endocyte/Novartis, Basel, Switzerland) are, to our knowledge, not yet tested in humans, they are also progressing towards clinical translation with EC17 (folate-FITC) in osteosarcoma patients [99]. At the end of 2019, Aleta Biotherapeutics announced that the good manufacturing practice (GMP) production of the bifunctional adaptor molecule CD19ECD-*α*CD20 had been initiated. Following the IMPACT strategy, it is planned to use this fusion protein for the reactivation of persistent CD19 CAR T cells in patients that relapsed with CD19^neg^ disease after CD19 CAR T cell therapy [83].

## 7. Future Directions of Adaptor CARs

Except for effector T cells, also other immune cells can be engineered to express adaptor CARs, and therefore be redirected to any desired target antigen. The second most promising effector cell population with a high lytic capacity are natural killer (NK) cells. Their utility for CAR approaches is underlined by the recent results of a phase 1/2 clinical trial on non-HLA-matched CD19 CAR NK cells in B cell lymphoma and leukemia patients [100]. Seven out of 11 patients experienced complete remissions without the development of CRS, CRES, or graft-versus-host disease (GvHD). To our knowledge, adaptor CAR approaches have not yet been applied in primary NK cells, but two reports on the clinically applicable cell line NK-92 exist [64,101]. In 2015, Clémenceau and colleagues genetically modified NK-92 cells with CD16-Fc*ε*RI*γ* adaptor CARs [101]. In the presence of trastuzumab, they were able to control the growth of HER2^pos^ tumors in experimental mice. More recently, Mitwasi et al. presented results on universal UniCAR NK-92 cells [64]. Upon cross-linkage via short-living or extended half-life TMs, GD2^pos^ tumors were efficiently eliminated both in vitro and in vivo. As the clinical safety of irradiated NK-92 cells was already proven [102,103,104,105], the adaptor CAR NK cells might represent a promising universal off-the-shelf cellular product that can be broadly applied to any cancer patient in combination with the desired adaptor molecule mixture.

Given their phagocytotic activity and ability to efficiently penetrate solid tumors, macrophages emerged as another promising effector cell population [106]. The safety and feasibility of adoptive macrophage transfer have been shown in patients with solid tumors, although only minor anti-tumor responses could be observed [107,108]. In order to boost therapeutic effects, Klichinsky and colleagues recently successfully engineered primary human macrophages to express conventional Her2-specific CARs [106]. In vitro and in vivo studies demonstrated that their phagocytotic activity was efficiently redirected against solid tumor cells in a strict antigen-specific manner. Most importantly, adenoviral transduction shifted human primary macrophages towards a pro-inflammatory phenotype that was maintained within the human TME, including in the presence of immunosuppressive M2 macrophages. Moreover, CAR macrophages were able to stimulate other immune effector cells of the TME, e.g., attracting resting and activated T cells. As shown in experimental mice, this resulted in synergistic therapeutic effects of CAR macrophages and polyclonal T cells. Overall, these data also encourage the engineering of controllable adaptor CAR macrophages that can be flexibly redirected to any desired tumor antigen. In combination with adaptor CAR T cells, they might be able to overcome major hurdles of solid tumor therapy, such as the immunosuppressive TME and poor tumor infiltration of effector CAR (T) cells. Nonetheless, it remains unclear whether “inactive” adaptor CAR macrophages can maintain their pro-inflammatory signature also in the absence of adaptor molecules or whether they then turn into immunosuppressive M2 macrophages, counteracting efficient treatment (see [106]). 

Due to their immunosuppressive functions, adoptively transferred polyclonal Tregs are being tested to treat inflammation-related diseases, such as GvHD [109,110] and autoimmune disorders [111,112]. However, the poor trafficking of polyclonal Tregs into inflamed tissues emphasizes the clinical need for the site-specific redirection of Tregs. Thus, Tregs-expressing adaptor CARs will open a completely new field of application beyond cancer [113,114]. In 2018, Koristka et al. demonstrated the feasibility of this approach by applying the UniCAR platform technology for the retargeting of human Tregs [115]. Tregs from patients with GvHD or multiple sclerosis were successfully modified with UniCARs and substantially suppressed patient-derived effector T cells. From a safety perspective, UniCARs with CD137-CD3ζ-signaling domains were more suitable for Treg manipulation compared to UniCARs providing CD28 costimulation [115]. Taken together, adaptor CAR Tregs are promising candidates to locally suppress inflammatory responses with high flexibility and safety. The most challenging aspect for their clinical application is the selection of suitable target antigens.

## 8. Conclusions

Switchable adaptor CAR systems present novel and promising solutions to key problems of conventional CAR technology. This is underlined by the rapid preclinical development of various different platforms, which all have their inherent advantages and disadvantages. At present, the first phase 1/2 trials are underway, which will challenge these novel tools with regard to their applicability in the clinical setting. The employment of one single CAR T cell product facilitates the development of standardized protocols for vector design, T cell engineering, and therapy. However, the technology will also have to face and address common hurdles of immunotherapies, like pre-existing T cell defects, T cell exhaustion, and poor T cell proliferation and persistence, but can benefit from the vast experience gained with conventional CARs so far.

## Figures and Tables

**Table 1:** Clinical trials of adaptor CAR T cells in the United States and Europe. ACTR: antibody-coupled T cell receptor; TBA: to be announced.

Disease	Age Group (years)	Adaptor CAR	Adaptor Molecule	Location Countries	ClinicalTrial.Gov Reference Number
refractory or relapsed CD20^pos^ B cell lymphoma	18–75	CD16-BB/ζ (ACTR087)	rituximab	United States	NCT02776813 (completed) 08/2016-02/2020
refractory or relapsed CD20^pos^ B cell lymphoma	18–80	CD16-28 (ACTR707)	rituximab	United States	NCT03189836 (active) start: 06/2017
relapsed or refractory multiple myeloma	18–80	ACTR087	SEA-BCMA	United States	NCT03266692 (terminated) 02/2018-10/2019
HER2^pos^ advanced malignancies	18–75	ACTR087 ACTR707	trastuzumab	United States	NCT03680560 (terminated) 03/2019-03/2020
B cell lymphomas multiple myeloma HER2^pos^ solid tumors	≥18	ACTR087 ACTR707	Trastuzumab rituximab SEA-BCMA	United States	NCT02840110 (enrolling by invitation) start: 10/2016
CD123^pos^ hematologic and lymphatic malignancies	≥18	UniCAR-28/ζ (UniCAR02-T)	CD123 TM (TM123)	Germany	NCT04230265 (recruiting) start: 01/2020
hematologic malignancies	TBA	*α*-PNE CAR (CLBR001)	CD19 Fab switch (SWI019)	TBA	TBA start: 2020

## References

[1] Eshhar Z. (2008). The T-body approach: Redirecting T cells with antibody specificity. Handb. Exp. Pharmacol..

[2] MacKay M., Afshinnekoo E., Rub J., Hassan C., Khunte M., Baskaran N., Owens B., Liu L., Roboz G.J., Guzman M.L. (2020). The therapeutic landscape for cells engineered with chimeric antigen receptors. Nat. Biotechnol..

[3] Freyer C.W. (2018). Tisagenlecleucel: The First CAR on the Highway to Remission for Acute Lymphoblastic Leukemia. J. Adv. Pract. Oncol..

[4] Bouchkouj N., Kasamon Y.L., de Claro R.A., George B., Lin X., Lee S., Blumenthal G.M., Bryan W., McKee A.E., Pazdur R. (2019). FDA Approval Summary: Axicabtagene Ciloleucel for Relapsed or Refractory Large B-cell Lymphoma. Clin. Cancer Res..

[5] Ali S., Kjeken R., Niederlaender C., Markey G., Saunders T.S., Opsata M., Moltu K., Bremnes B., Grønevik E., Muusse M. (2020). The European Medicines Agency Review of Kymriah (Tisagenlecleucel) for the Treatment of Acute Lymphoblastic Leukemia and Diffuse Large B-Cell Lymphoma. Oncologist.

[6] Fry T.J., Shah N.N., Orentas R.J., Stetler-Stevenson M., Yuan C.M., Ramakrishna S., Wolters P., Martin S., Delbrook C., Yates B. (2018). CD22-targeted CAR T cells induce remission in B-ALL that is naive or resistant to CD19-targeted CAR immunotherapy. Nat. Med..

[7] Ali S.A., Shi V., Maric I., Wang M., Stroncek D.F., Rose J.J., Brudno J.N., Stetler-Stevenson M., Feldman S.A., Hansen B.G. (2016). T cells expressing an anti-B-cell maturation antigen chimeric antigen receptor cause remissions of multiple myeloma. Blood.

[8] Raje N., Berdeja J., Lin Y., Siegel D., Jagannath S., Madduri D., Liedtke M., Rosenblatt J., Maus M.V., Turka A. (2019). Anti-BCMA CAR T-Cell Therapy bb2121 in Relapsed or Refractory Multiple Myeloma. N. Engl. J. Med..

[9] Sotillo E., Barrett D.M., Black K.L., Bagashev A., Oldridge D., Wu G., Sussman R., Lanauze C., Ruella M., Gazzara M.R. (2015). Convergence of Acquired Mutations and Alternative Splicing of CD19 Enables Resistance to CART-19 Immunotherapy. Cancer Discov..

[10] Brown C.E., Alizadeh D., Starr R., Weng L., Wagner J.R., Naranjo A., Ostberg J.R., Blanchard M.S., Kilpatrick J., Simpson J. (2016). Regression of Glioblastoma after Chimeric Antigen Receptor T-Cell Therapy. N. Engl. J. Med..

[11] O’Rourke D.M., Nasrallah M.P., Desai A., Melenhorst J.J., Mansfield K., Morrissette J.J.D., Martinez-Lage M., Brem S., Maloney E., Shen A. (2017). A single dose of peripherally infused EGFRvIII-directed CAR T cells mediates antigen loss and induces adaptive resistance in patients with recurrent glioblastoma. Sci. Transl. Med..

[12] Shalabi H., Kraft I.L., Wang H.W., Yuan C.M., Yates B., Delbrook C., Zimbelman J.D., Giller R., Stetler-Stevenson M., Jaffe E.S. (2018). Sequential loss of tumor surface antigens following chimeric antigen receptor T-cell therapies in diffuse large B-cell lymphoma. Haematologica.

[13] Fesnak A.D., June C.H., Levine B.L. (2016). Engineered T cells: The promise and challenges of cancer immunotherapy. Nat. Rev. Cancer.

[14] Morgan R.A., Yang J.C., Kitano M., Dudley M.E., Laurencot C.M., Rosenberg S.A. (2010). Case Report of a Serious Adverse Event Following the Administration of T Cells Transduced With a Chimeric Antigen Receptor Recognizing ERBB2. Mol. Ther..

[15] Lee D.W., Santomasso B.D., Locke F.L., Ghobadi A., Turtle C.J., Brudno J.N., Maus M.V., Park J.H., Mead E., Pavletic S. (2019). ASTCT Consensus Grading for Cytokine Release Syndrome and Neurologic Toxicity Associated with Immune Effector Cells. Biol. Blood Marrow. Transplant..

[16] Gill S., Maus M.V., Porter D.L. (2016). Chimeric antigen receptor T cell therapy: 25years in the making. Blood Rev..

[17] Cartellieri M., Bachmann M., Feldmann A., Bippes C., Stamova S., Wehner R., Temme A., Schmitz M. (2010). Chimeric antigen receptor-engineered T cells for immunotherapy of cancer. J. Biomed. Biotechnol..

[18] Tokarew N., Ogonek J., Endres S., von Bergwelt-Baildon M., Kobold S. (2019). Teaching an old dog new tricks: Next-generation CAR T cells. Br. J. Cancer..

[19] Hoyos V., Savoldo B., Quintarelli C., Mahendravada A., Zhang M., Vera J., Heslop H.E., Rooney C.M., Brenner M.K., Dotti G. (2010). Engineering CD19-specific T lymphocytes with interleukin-15 and a suicide gene to enhance their anti-lymphoma/leukemia effects and safety. Leukemia.

[20] Budde L.E., Berger C., Lin Y., Wang J., Lin X., Frayo S.E., Brouns S.A., Spencer D.M., Till B.G., Jensen M.C. (2013). Combining a CD20 chimeric antigen receptor and an inducible caspase 9 suicide switch to improve the efficacy and safety of T cell adoptive immunotherapy for lymphoma. PLoS ONE.

[21] Diaconu I., Ballard B., Zhang M., Chen Y., West J., Dotti G., Savoldo B. (2017). Inducible Caspase-9 Selectively Modulates the Toxicities of CD19-Specific Chimeric Antigen Receptor-Modified T Cells. Mol. Ther..

[22] Larson S.M., Truscott L.C., Chiou T.T., Patel A., Kao R., Tu A., Tyagi T., Lu X., Elashoff D., De Oliveira S.N. (2017). Pre-clinical development of gene modification of haematopoietic stem cells with chimeric antigen receptors for cancer immunotherapy. Hum. Vaccines Immunother..

[23] Vogler I., Newrzela S., Hartmann S., Schneider N., von Laer D., Koehl U., Grez M. (2010). An improved bicistronic CD20/tCD34 vector for efficient purification and in vivo depletion of gene-modified T cells for adoptive immunotherapy. Mol. Ther..

[24] Philip B., Kokalaki E., Mekkaoui L., Thomas S., Straathof K., Flutter B., Marin V., Marafioti T., Chakraverty R., Linch D. (2014). A highly compact epitope-based marker/suicide gene for easier and safer T-cell therapy. Blood.

[25] Wang X., Chang W.C., Wong C.W., Colcher D., Sherman M., Ostberg J.R., Forman S.J., Riddell S.R., Jensen M.C. (2011). A transgene-encoded cell surface polypeptide for selection, in vivo tracking, and ablation of engineered cells. Blood.

[26] Paszkiewicz P.J., Fräßle S.P., Srivastava S., Sommermeyer D., Hudecek M., Drexler I., Sadelain M., Liu L., Jensen M.C., Riddell S.R. (2016). Targeted antibody-mediated depletion of murine CD19 CAR T cells permanently reverses B cell aplasia. J. Clin. Investig..

[27] Clémenceau B., Congy-Jolivet N., Gallot G., Vivien R., Gaschet J., Thibault G., Vié H. (2006). Antibody-dependent cellular cytotoxicity (ADCC) is mediated by genetically modified antigen-specific human T lymphocytes. Blood.

[28] Ochi F., Fujiwara H., Tanimoto K., Asai H., Miyazaki Y., Okamoto S., Mineno J., Kuzushima K., Shiku H., Barrett J. (2014). Gene-modified human α/β-T cells expressing a chimeric CD16-CD3ζ receptor as adoptively transferable effector cells for anticancer monoclonal antibody therapy. Cancer Immunol. Res..

[29] Tanaka H., Fujiwara H., Ochi F., Tanimoto K., Casey N., Okamoto S., Mineno J., Kuzushima K., Shiku H., Sugiyama T. (2016). Development of Engineered T Cells Expressing a Chimeric CD16-CD3ζ Receptor to Improve the Clinical Efficacy of Mogamulizumab Therapy Against Adult T-Cell Leukemia. Clin. Cancer Res..

[30] Kudo K., Imai C., Lorenzini P., Kamiya T., Kono K., Davidoff A.M., Chng W.J., Campana D. (2014). T lymphocytes expressing a CD16 signaling receptor exert antibody-dependent cancer cell killing. Cancer Res..

[31] Rataj F., Jacobi S.J., Stoiber S., Asang F., Ogonek J., Tokarew N., Cadilha B.L., van Puijenbroek E., Heise C., Duewell P. (2019). High-affinity CD16-polymorphism and Fc-engineered antibodies enable activity of CD16-chimeric antigen receptor-modified T cells for cancer therapy. Br. J. Cancer.

[32] Caratelli S., Arriga R., Sconocchia T., Ottaviani A., Lanzilli G., Pastore D., Cenciarelli C., Venditti A., Del Principe M.I., Lauro D. (2020). In vitro elimination of epidermal growth factor receptor-overexpressing cancer cells by CD32A-chimeric receptor T cells in combination with cetuximab or panitumumab. Int. J. Cancer.

[33] Goulet D.R., Atkins W.M. (2020). Considerations for the Design of Antibody-Based Therapeutics. J. Pharm. Sci..

[34] Strohl W.R. (2018). Current progress in innovative engineered antibodies. Protein Cell..

[35] Urbanska K., Lanitis E., Poussin M., Lynn R., Gavin B.P., Kelderman S., Yu J., Scholler N., Powell D.J. (2012). A universal strategy for adoptive immunotherapy of cancer through use of a novel T cell antigen receptor. Cancer Res..

[36] Lohmueller J.J., Ham J.D., Kvorjak M., Finn O.J. (2017). mSA2 affinity-enhanced biotin-binding CAR T cells for universal tumor targeting. Oncoimmunology.

[37] Stratton S.L., Horvath T.D., Bogusiewicz A., Matthews N.I., Henrich C.L., Spencer H.J., Moran J.H., Mock D.M. (2010). Plasma concentration of 3-hydroxyisovaleryl carnitine is an early and sensitive indicator of marginal biotin deficiency in humans. Am. J. Clin. Nutr..

[38] Dale G.L., Gaddy P., Pikul F.J. (1994). Antibodies against biotinylated proteins are present in normal human serum. J. Lab. Clin. Med..

[39] Paganelli G., Magnani P., Zito F., Villa E., Sudati F., Lopalco L., Rossetti C., Malcovati M., Chiolerio F., Seccamani E. (1991). Three-step monoclonal antibody tumor targeting in carcinoembryonic antigen-positive patients. Cancer Res..

[40] Paganelli G., Chinol M., Maggiolo M., Sidoli A., Corti A., Baroni S., Siccardi A.G. (1997). The three-step pretargeting approach reduces the human anti-mouse antibody response in patients submitted to radioimmunoscintigraphy and radioimmunotherapy. Eur. J. Nucl. Med..

[41] Tamada K., Geng D., Sakoda Y., Bansal N., Srivastava R., Li Z., Davila E. (2012). Redirecting gene-modified T cells toward various cancer types using tagged antibodies. Clin. Cancer Res..

[42] Kim M.S., Ma J.S., Yun H., Cao Y., Kim J.Y., Chi V., Wang D., Woods A., Sherwood L., Caballero D. (2015). Redirection of genetically engineered CAR-T cells using bifunctional small molecules. J. Am. Chem. Soc..

[43] Cao Y., Rodgers D.T., Du J., Ahmad I., Hampton E.N., Ma J.S., Mazagova M., Choi S.H., Yun H.Y., Xiao H. (2016). Design of Switchable Chimeric Antigen Receptor T Cells Targeting Breast Cancer. Angew. Chem. Int. Ed. Engl..

[44] Ma J.S., Kim J.Y., Kazane S.A., Choi S.H., Yun H.Y., Kim M.S., Rodgers D.T., Pugh H.M., Singer O., Sun S.B. (2016). Versatile strategy for controlling the specificity and activity of engineered T cells. Proc. Natl. Acad. Sci. USA.

[45] Zhang E., Gu J., Xue J., Lin C., Liu C., Li M., Hao J., Setrerrahmane S., Chi X., Qi W. (2018). Accurate control of dual-receptor-engineered T cell activity through a bifunctional anti-angiogenic peptide. J. Hematol. Oncol..

[46] Chu W., Zhou Y., Tang Q., Wang M., Ji Y., Yan J., Yin D., Zhang S., Lu H., Shen J. (2018). Bi-specific ligand-controlled chimeric antigen receptor T-cell therapy for non-small cell lung cancer. Biosci. Trends.

[47] Lee Y.G., Chu H., Lu Y., Leamon C.P., Srinivasarao M., Putt K.S., Low P.S. (2019). Regulation of CAR T cell-mediated cytokine release syndrome-like toxicity using low molecular weight adapters. Nat. Commun..

[48] Lee Y.G., Marks I., Srinivasarao M., Kanduluru A.K., Mahalingam S.M., Liu X., Chu H., Low P.S. (2019). Use of a Single CAR T Cell and Several Bispecific Adapters Facilitates Eradication of Multiple Antigenically Different Solid Tumors. Cancer Res..

[49] Lu Y.J., Chu H., Wheeler L.W., Nelson M., Westrick E., Matthaei J.F., Cardle I.I., Johnson A., Gustafson J., Parker N. (2019). Preclinical Evaluation of Bispecific Adaptor Molecule Controlled Folate Receptor CAR-T Cell Therapy With Special Focus on Pediatric Malignancies. Front. Oncol..

[50] Van Dam G.M., Themelis G., Crane L.M., Harlaar N.J., Pleijhuis R.G., Kelder W., Sarantopoulos A., de Jong J.S., Arts H.J., van der Zee A.G. (2011). Intraoperative tumor-specific fluorescence imaging in ovarian cancer by folate receptor-α targeting: First in-human results. Nat. Med..

[51] Koristka S., Cartellieri M., Feldmann A., Arndt C., Loff S., Michalk I., Aliperta R., von Bonin M., Bornhäuser M., Ehninger A. (2014). Flexible antigen-specific redirection of human regulatory T cells via a novel universal chimeric antigen receptor system. Blood.

[52] Cartellieri M., Loff S., von Bonin M., Bejestani E.P., Ehninger A., Feldmann A., Koristka S., Arndt C., Ehninger G., Bachmann M.P. (2015). Unicar: A Novel Modular Retargeting Platform Technology for CAR T Cells. Blood.

[53] Carmo-Fonseca M., Pfeifer K., Schröder H.C., Vaz M.F., Fonseca J.E., Müller W.E.G., Bachmann M. (1989). Identification of La ribonucleoproteins as a component of interchromatin granules. Exp. Cell. Res..

[54] Koristka S., Cartellieri M., Arndt C., Bippes C.C., Feldmann A., Michalk I., Wiefel K., Stamova S., Schmitz M., Ehninger G. (2013). Retargeting of regulatory T cells to surface-inducible autoantigen La/SS-B. J. Autoimmun..

[55] Cartellieri M., Feldmann A., Koristka S., Arndt C., Loff S., Ehninger A., von Bonin M., Bejestani E.P., Ehninger G., Bachmann M.P. (2016). Switching CAR T cells on and off: A novel modular platform for retargeting of T cells to AML blasts. Blood Cancer J..

[56] Feldmann A., Arndt C., Bergmann R., Loff S., Cartellieri M., Bachmann D., Aliperta R., Hetzenecker M., Ludwig F., Albert S. (2017). Retargeting of T lymphocytes to PSCA- or PSMA positive prostate cancer cells using the novel modular chimeric antigen receptor platform technology "UniCAR". Oncotarget.

[57] Albert S., Arndt C., Feldmann A., Bergmann R., Bachmann D., Koristka S., Ludwig F., Ziller-Walter P., Kegler A., Gärtner S. (2017). A novel nanobody-based target module for retargeting of T lymphocytes to EGFR-expressing cancer cells via the modular UniCAR platform. Oncoimmunology.

[58] Mitwasi N., Feldmann A., Bergmann R., Berndt N., Arndt C., Koristka S., Kegler A., Jureczek J., Hoffmann A., Ehninger A. (2017). Development of novel target modules for retargeting of UniCAR T cells to GD2 positive tumor cells. Oncotarget.

[59] Bachmann D., Aliperta R., Bergmann R., Feldmann A., Koristka S., Arndt C., Loff S., Welzel P., Albert S., Kegler A. (2018). Retargeting of UniCAR T cells with an in vivo synthesized target module directed against CD19 positive tumor cells. Oncotarget.

[60] Albert S., Arndt C., Koristka S., Berndt N., Bergmann R., Feldmann A., Schmitz M., Pietzsch J., Steinbach J., Bachmann M. (2018). From mono- to bivalent: Improving theranostic properties of target modules for redirection of UniCAR T cells against EGFR-expressing tumor cells in vitro and in vivo. Oncotarget.

[61] Loureiro L.R., Feldmann A., Bergmann R., Koristka S., Berndt N., Arndt C., Pietzsch J., Novo C., Videira P., Bachmann M. (2018). Development of a novel target module redirecting UniCAR T cells to Sialyl Tn-expressing tumor cells. Blood Cancer J..

[62] Arndt C., Feldmann A., Koristka S., Schäfer M., Bergmann R., Mitwasi N., Berndt N., Bachmann D., Kegler A., Schmitz M. (2019). A theranostic PSMA ligand for PET imaging and retargeting of T cells expressing the universal chimeric antigen receptor UniCAR. OncoImmunology.

[63] Arndt C., Loureiro L.R., Feldmann A., Jureczek J., Bergmann R., Máthé D., Hegedüs N., Berndt N., Koristka S., Mitwasi N. (2020). UniCAR T cell immunotherapy enables efficient elimination of radioresistant cancer cells. OncoImmunology.

[64] Mitwasi N., Feldmann A., Arndt C., Koristka S., Berndt N., Jureczek J., Loureiro L.R., Bergmann R., Máthé D., Hegedüs N. (2020). “UniCAR”-modified off-the-shelf NK-92 cells for targeting of GD2-expressing tumour cells. Sci. Rep..

[65] Loureiro L.R., Feldmann A., Bergmann R., Koristka S., Berndt N., Máthé D., Hegedüs N., Szigeti K., Videira P.A., Bachmann M. (2020). Extended half-life target module for sustainable UniCAR T-cell treatment of STn-expressing cancers. J. Exp. Clin. Cancer Res..

[66] Koristka S., Ziller-Walter P., Bergmann R., Arndt C., Feldmann A., Kegler A., Cartellieri M., Ehninger A., Ehninger G., Bornhäuser M. (2019). Anti-CAR-engineered T cells for epitope-based elimination of autologous CAR T cells. Cancer Immunol. Immunother..

[67] Tröster H., Metzger T.E., Semsei I., Schwemmle M., Winterpacht A., Zabel B., Bachmann M. (1994). One gene, two transcripts: Isolation of an alternative transcript encoding for the autoantigen La/SS-B from a cDNA library of a patient with primary Sjögrens’ syndrome. J. Exp. Med..

[68] Yiannaki E.E., Tzioufas A.G., Bachmann M., Hantoumi J., Tsikaris V., Sakarellos-Daitsiotis M., Sakarellos C., Moutsopoulos H.M. (1998). The value of synthetic linear epitope analogues of La/SSB for the detection of autoantibodies to La/SSB; specificity, sensitivity and comparison of methods. Clin. Exp. Immunol..

[69] Tröster H., Bartsch H., Klein R., Metzger T.E., Pollak G., Semsei I., Schwemmle M., Pruijn G.J., van Venrooij W.J., Bachmann M. (1995). Activation of a murine autoreactive B cell by immunization with human recombinant autoantigen La/SS-B: Characterization of the autoepitope. J. Autoimmun..

[70] Dudek N.L., Maier S., Chen Z.J., Mudd P.A., Mannering S.I., Jackson D.C., Zeng W., Keech C.L., Hamlin K., Pan Z.J. (2007). T cell epitopes of the La/SSB autoantigen in humanized transgenic mice expressing the HLA class II haplotype DRB1*0301/DQB1*0201. Arthritis. Rheum..

[71] Bachmann M. (2019). The UniCAR system: A modular CAR T cell approach to improve the safety of CAR T cells. Immunol. Lett..

[72] Rodgers D.T., Mazagova M., Hampton E.N., Cao Y., Ramadoss N.S., Hardy I.R., Schulman A., Du J., Wang F., Singer O. (2016). Switch-mediated activation and retargeting of CAR-T cells for B-cell malignancies. Proc. Natl. Acad. Sci. USA.

[73] Raj D., Yang M.H., Rodgers D., Hampton E.N., Begum J., Mustafa A., Lorizio D., Garces I., Propper D., Kench J.G. (2019). Switchable CAR-T cells mediate remission in metastatic pancreatic ductal adenocarcinoma. Gut.

[74] Viaud S., Ma J.S.Y., Hardy I.R., Hampton E.N., Benish B., Sherwood L., Nunez V., Ackerman C.J., Khialeeva E., Weglarz M. (2018). Switchable control over in vivo CAR T expansion, B cell depletion, and induction of memory. Proc. Natl. Acad. Sci. USA.

[75] Cho J.H., Collins J.J., Wong W.W. (2018). Universal Chimeric Antigen Receptors for Multiplexed and Logical Control of T Cell Responses. Cell.

[76] Minutolo N.G., Sharma P., Poussin M., Shaw L.C., Brown D.P., Hollander E.E., Smole A., Rodriguez-Garcia A., Hui J.Z., Zappala F. (2020). Quantitative Control of Gene-Engineered T-Cell Activity through the Covalent Attachment of Targeting Ligands to a Universal Immune Receptor. J. Am. Chem. Soc..

[77] Li L., Fierer J.O., Rapoport T.A., Howarth M. (2014). Structural analysis and optimization of the covalent association between SpyCatcher and a peptide Tag. J. Mol. Biol..

[78] Stamova S., Koristka S., Keil J., Arndt C., Feldmann A., Michalk I., Bartsch H., Bippes C.C., Schmitz M., Cartellieri M. (2012). Cancer immunotherapy by retargeting of immune effector cells via recombinant bispecific antibody constructs. Antibodies.

[79] Urbanska K., Lynn R.C., Stashwick C., Thakur A., Lum L.G., Powell D.J. (2014). Targeted cancer immunotherapy via combination of designer bispecific antibody and novel gene-engineered T cells. J. Transl. Med..

[80] Karches C.H., Benmebarek M.R., Schmidbauer M.L., Kurzay M., Klaus R., Geiger M., Rataj F., Cadilha B.L., Lesch S., Heise C. (2019). Bispecific Antibodies Enable Synthetic Agonistic Receptor-Transduced T Cells for Tumor Immunotherapy. Clin. Cancer Res..

[81] Ambrose C., Su L., Wu L., Lobb R.R., Rennert P.D. (2017). Abstract 3768: CAR T cells specific for CD19 can be redirected to kill CD19 negative tumors. Cancer Res..

[82] Klesmith J.R., Su L., Wu L., Schrack I.A., Dufort F.J., Birt A., Ambrose C., Hackel B.J., Lobb R.R., Rennert P.D. (2019). Retargeting CD19 Chimeric Antigen Receptor T Cells via Engineered CD19-Fusion Proteins. Mol. Pharm..

[83] Rennert P., Su L., Dufort F., Birt A., Sanford T., Wu L., Ambrose C., Lobb R. (2019). A Novel CD19-Anti-CD20 Bridging Protein Prevents and Reverses CD19-Negative Relapse from CAR19 T Cell Treatment In Vivo. Blood.

[84] Jureczek J., Bergmann R., Berndt N., Koristka S., Kegler A., Puentes-Cala E., Soto J.A., Arndt C., Bachmann M., Feldmann A. (2019). An oligo-His-tag of a targeting module does not influence its biodistribution and the retargeting capabilities of UniCAR T cells. Sci. Rep..

[85] Flanagan R.J., Jones A.L. (2004). Fab antibody fragments: Some applications in clinical toxicology. Drug. Saf..

[86] Wang W., Wang E.Q., Balthasar J.P. (2008). Monoclonal antibody pharmacokinetics and pharmacodynamics. Clin. Pharmacol. Ther..

[87] Lu Y., Xu L.C., Parker N., Westrick E., Reddy J.A., Vetzel M., Low P.S., Leamon C.P. (2006). Preclinical pharmacokinetics, tissue distribution, and antitumor activity of a folate-hapten conjugate-targeted immunotherapy in hapten-immunized mice. Mol. Cancer Ther..

[88] Fasslrinner F., Arndt C., Koristka S., Feldmann A., Altmann H., von Bonin M., Schmitz M., Bornhäuser M., Bachmann M. (2019). Midostaurin abrogates CD33-directed UniCAR and CD33-CD3 bispecific antibody therapy in acute myeloid leukaemia. Br. J. Haematol..

[89] Ghosh A., Heston W.D. (2004). Tumor target prostate specific membrane antigen (PSMA) and its regulation in prostate cancer. J. Cell. Biochem..

[90] Silver D.A., Pellicer I., Fair W.R., Heston W.D., Cordon-Cardo C. (1997). Prostate-specific membrane antigen expression in normal and malignant human tissues. Clin. Cancer Res..

[91] Lal A., Peters H., St Croix B., Haroon Z.A., Dewhirst M.W., Strausberg R.L., Kaanders J.H., van der Kogel A.J., Riggins G.J. (2001). Transcriptional response to hypoxia in human tumors. J. Natl. Cancer Inst..

[92] Puig-Kröger A., Sierra-Filardi E., Domínguez-Soto A., Samaniego R., Corcuera M.T., Gómez-Aguado F., Ratnam M., Sánchez-Mateos P., Corbí A.L. (2009). Folate receptor beta is expressed by tumor-associated macrophages and constitutes a marker for M2 anti-inflammatory/regulatory macrophages. Cancer Res..

[93] Kegler A., Koristka S., Bergmann R., Berndt N., Arndt C., Feldmann A., Hoffmann A., Bornhäuser M., Schmitz M., Bachmann M.P. (2019). T cells engrafted with a UniCAR 28/z outperform UniCAR BB/z-transduced T cells in the face of regulatory T cell-mediated immunosuppression. Oncoimmunology.

[94] Loff S., Meyer J.-E., Dietrich J., Spehr J., Riewaldt J., von Bonin M., Gründer C., Franke K., Feldmann A., Bachmann M. (2018). Late-Stage Preclinical Characterization of Switchable CD123-Specific CAR-T for Treatment of Acute Leukemia. Blood.

[95] Minutolo N.G., Hollander E.E., Powell D.J. (2019). The Emergence of Universal Immune Receptor T Cell Therapy for Cancer. Front. Oncol..

[96] FDA Places Clinical Hold on Unum Therapeutics Phase 1 Trial Evaluating ACTR087 for Relapsed/Refractory CD20+ B Cell Non-Hodgkin Lymphoma. https://www.trialsitenews.com/fda-places-clinical-hold-on-unum-therapeutics-phase-1-trial-evaluating-actr087-for-relapsed-refractory-cd20-b-cell-non-hodgkin-lymphoma/.

[97] Unum Therapeutics Provides Updates to its Phase 1 Trial of ACTR707 for HER2+ Solid Tumor Cancers. https://www.globenewswire.com/news-release/2020/01/29/1976692/0/en/Unum-Therapeutics-Provides-Updates-to-its-Phase-1-Trial-of-ACTR707-for-HER2-Solid-Tumor-Cancers.html.

[98] AbbVie & Scripps-based Calibr Moves Novel ‘Switchable’ CAR-T Technology to Phase I Clinical Trial. https://www.trialsitenews.com/abbvie-scripps-based-calibr-moves-novel-switchable-car-t-technology-to-phase-i-clinical-trial/.

[99] Endocyte Provides Third Quarter 2018 Financial Results and Operational Update. https://www.globenewswire.com/news-release/2018/11/07/1647347/0/en/Endocyte-Provides-Third-Quarter-2018-Financial-Results-and-Operational-Update.html.

[100] Liu E., Marin D., Banerjee P., Macapinlac H.A., Thompson P., Basar R., Nassif Kerbauy L., Overman B., Thall P., Kaplan M. (2020). Use of CAR-Transduced Natural Killer Cells in CD19-Positive Lymphoid Tumors. N. Engl. J. Med..

[101] Clémenceau B., Valsesia-Wittmann S., Jallas A.C., Vivien R., Rousseau R., Marabelle A., Caux C., Vié H. (2015). In Vitro and In Vivo Comparison of Lymphocytes Transduced with a Human CD16 or with a Chimeric Antigen Receptor Reveals Potential Off-Target Interactions due to the IgG2 CH2-CH3 CAR-Spacer. J. Immunol. Res..

[102] Tonn T., Schwabe D., Klingemann H.G., Becker S., Esser R., Koehl U., Suttorp M., Seifried E., Ottmann O.G., Bug G. (2013). Treatment of patients with advanced cancer with the natural killer cell line NK-92. Cytotherapy.

[103] Arai S., Meagher R., Swearingen M., Myint H., Rich E., Martinson J., Klingemann H. (2008). Infusion of the allogeneic cell line NK-92 in patients with advanced renal cell cancer or melanoma: A phase I trial. Cytotherapy.

[104] Williams B.A., Law A.D., Routy B., denHollander N., Gupta V., Wang X.H., Chaboureau A., Viswanathan S., Keating A. (2017). A phase I trial of NK-92 cells for refractory hematological malignancies relapsing after autologous hematopoietic cell transplantation shows safety and evidence of efficacy. Oncotarget.

[105] Boyiadzis M., Agha M., Redner R.L., Sehgal A., Im A., Hou J.Z., Farah R., Dorritie K.A., Raptis A., Lim S.H. (2017). Phase 1 clinical trial of adoptive immunotherapy using “off-the-shelf” activated natural killer cells in patients with refractory and relapsed acute myeloid leukemia. Cytotherapy.

[106] Klichinsky M., Ruella M., Shestova O., Lu X.M., Best A., Zeeman M., Schmierer M., Gabrusiewicz K., Anderson N.R., Petty N.E. (2020). Human chimeric antigen receptor macrophages for cancer immunotherapy. Nat. Biotechnol..

[107] Andreesen R., Hennemann B., Krause S.W. (1998). Adoptive immunotherapy of cancer using monocyte-derived macrophages: Rationale, current status, and perspectives. J. Leukoc. Biol..

[108] Burger M., Thiounn N., Denzinger S., Kondas J., Benoit G., Chapado M.S., Jimenz-Cruz F.J., Kisbenedek L., Szabo Z., Zsolt D. (2010). The application of adjuvant autologous antravesical macrophage cell therapy vs. BCG in non-muscle invasive bladder cancer: A multicenter, randomized trial. J. Transl. Med..

[109] Brunstein C.G., Miller J.S., Cao Q., McKenna D.H., Hippen K.L., Curtsinger J., Defor T., Levine B.L., June C.H., Rubinstein P. (2011). Infusion of ex vivo expanded T regulatory cells in adults transplanted with umbilical cord blood: Safety profile and detection kinetics. Blood.

[110] Di Ianni M., Falzetti F., Carotti A., Terenzi A., Castellino F., Bonifacio E., Del Papa B., Zei T., Ostini R.I., Cecchini D. (2011). Tregs prevent GVHD and promote immune reconstitution in HLA-haploidentical transplantation. Blood.

[111] Marek-Trzonkowska N., Myśliwiec M., Dobyszuk A., Grabowska M., Derkowska I., Juścińska J., Owczuk R., Szadkowska A., Witkowski P., Młynarski W. (2014). Therapy of type 1 diabetes with CD4(+)CD25(high)CD127-regulatory T cells prolongs survival of pancreatic islets - results of one year follow-up. Clin. Immunol..

[112] Bluestone J.A., Buckner J.H., Fitch M., Gitelman S.E., Gupta S., Hellerstein M.K., Herold K.C., Lares A., Lee M.R., Li K. (2015). Type 1 diabetes immunotherapy using polyclonal regulatory T cells. Sci. Transl. Med..

[113] Sicard A., Boardman D.A., Levings M.K. (2018). Taking regulatory T-cell therapy one step further. Curr. Opin. Organ. Transplant..

[114] Ferreira L.M.R., Muller Y.D., Bluestone J.A., Tang Q. (2019). Next-generation regulatory T cell therapy. Nat. Rev. Drug Discov..

[115] Koristka S., Kegler A., Bergmann R., Arndt C., Feldmann A., Albert S., Cartellieri M., Ehninger A., Ehninger G., Middeke J.M. (2018). Engrafting human regulatory T cells with a flexible modular chimeric antigen receptor technology. J. Autoimmun..

